# Isolation, purification and partial physicochemical characterization of a lectin in *Andira pisonis Mart *seed

**DOI:** 10.1186/1753-6561-8-S4-P226

**Published:** 2014-10-01

**Authors:** Claudia Figueiredo Lossio, Mayara Torquato Lima da Silva, Lia Monteiro Barbosa, Cleane Gomes Moreira, Thaiz Batista Azevedo Rangel Miguel, Antonia Samia Fernandes do Nascimento, Ivanice Bezerra da Silva, Kyria Santiago do Nascimento, Benildo Sousa Cavada

**Affiliations:** 1 Universidade Federal do Ceara, Fortaleza, CE, Brazil

## 

Lectins are ubiquitous proteins in nature, with non-immune origin, which have at least one non-catalytic domain that binds carbohydrates specifically and reversibly. They can be found in vegetables leaves, stems and seeds. The Dalbergieae tribe has lectins which have specificity for different carbohydrates and also have several biological activities such as induction of rat paw edema, release of chemotactic mediators by macrophages, vasorelaxant effect in rat aortas, among others. This study aimed to isolate, purify and physiochemically characterize a lectin found in seeds of *Andira pisonis Mart *(Dalbergieae). *Andira pisonis Mart *seeds were ground into a fine powder and subjected to total protein extraction in 1 M ammonium sulfate. Soluble proteins were subjected to hemagglutination activity quantification by the Bradford method and essays of hemagglutination inibition activity by sugar. The lectin from *Andira pisonis Mart *(APL) was purified by affinity chromatography on Sepharose- Mannose matrix eluted in 0.1 M glycine buffer pH 2.6 with 0.15 M NaCl. The eluted fraction was dialyzed against distilled water, lyophilized and subjected to ion exchange chromatography on HiTrap SP XL 01. APL was eluted on 20 mM sodium acetate buffer pH 4.5 gradient of 0-1M NaCl. APL hemagglutinated rabbit erythrocytes (enzymatically treated) and other lectins from the tribe Dalbergieae and showed specificity for mannose (25 mM). SDS-PAGE analysis showed that APL is composed of a major 34 kDa double band and a minor 8 and 9 kDa double band. APL showed thermostability at 60° C. Further studies are still needed in order to better physicochemically characterize this protein and study its biotechnological potential on the referred conditions of vasorelaxant effect and chemotatic mediator.

